# The Effect of Lower-Body Positive Pressure on the Cardiorespiratory Response at Rest and during Submaximal Running Exercise

**DOI:** 10.3389/fphys.2018.00034

**Published:** 2018-01-30

**Authors:** Frédéric Stucky, Jean-Marc Vesin, Bengt Kayser, Barbara Uva

**Affiliations:** ^1^Institute of Sport Sciences, University of Lausanne, Lausanne, Switzerland; ^2^Applied Signal Processing Group, École Polytechnique Fédérale de Lausanne, Lausanne, Switzerland

**Keywords:** anti-gravity treadmill, lower body positive pressure, exercise, venous return, cardiac output, oxygen uptake

## Abstract

Anti-gravity treadmills facilitate locomotion by lower-body positive pressure (LBPP). Effects on cardiorespiratory regulation are unknown. Healthy men (30 ± 8 y, 178.3 ± 5.7 cm, 70.3 ± 8.0 kg; mean ± SD) stood upright (*n* = 10) or ran (*n* = 9) at 9, 11, 13, and 15 km.h^−1^ (5 min stages) with LBPP (0, 15, 40 mmHg). Cardiac output (CO), stroke volume (SV), heart rate (HR), blood pressure (BP), peripheral resistance (PR), and oxygen uptake (VO_2_) were monitored continuously. During standing, LBPP increased SV [by +29 ± 13 (+41%) and +42 ± 15 (+60%) ml, at 15 and 40 mmHg, respectively (*p* < 0.05)] and decreased HR [by −15 ± 6 (−20%) and −22 ± 9 (−29%) bpm (*p* < 0.05)] resulting in a transitory increase in CO [by +1.6 ± 1.0 (+32%) and +2.0 ± 1.0 (+39%) l.min^−1^ (*p* < 0.05)] within the first seconds of LBPP. This was accompanied by a transitory decrease in end-tidal PO_2_ [by −5 ± 3 (−5%) and −10 ± 4 (−10%) mmHg (*p* < 0.05)] and increase in VO_2_ [by +66 ± 53 (+26%) and +116 ± 64 (+46%) ml.min^−1^ (*p* < 0.05)], suggesting increased venous return and pulmonary blood flow. The application of LBPP increased baroreflex sensitivity (BRS) [by +1.8 ± 1.6 (+18%) and +4.6 ± 3.7 (+47%) at 15 and 40 mmHg LBPP, respectively *P* < 0.05]. After reaching steady-state exercise CO vs. VO_2_ relationships remained linear with similar slope and intercept for each participant (mean *R*^2^ = 0.84 ± 0.13) while MAP remained unchanged. It follows that (1) LBPP affects cardiorespiratory integration at the onset of exercise; (2) at a given LBPP, once reaching steady-state exercise, the cardiorespiratory load is reduced proportionally to the lower metabolic demand resulting from the body weight support; (3) the balance between cardiovascular response, oxygen delivery to the exercising muscles and blood pressure regulation is maintained at exercise steady-state; and (4) changes in baroreflex sensitivity may be involved in the regulation of cardiovascular parameters during LBPP.

## Introduction

Anti-gravity treadmills are used as rehabilitation and training tools to facilitate locomotion in patients with reduced mobility and/or exercise intolerance. Unloading can be achieved by various means. One type uses a conventional treadmill enclosed in a flexible chamber that can be sealed at waist level and inflated at various levels of pressure in order to lift the participant, thus providing the desired amount of body-weight support (Hoffman and Donaghe, 2011). Apart from its weight-unloading properties, this mechanism also generates positive pressure around the lower body (LBPP). By compressing the venous system of the lower limbs, LBPP may produce a translocation of blood toward the central circulation, increasing venous return and therefore lead to changes in cardiovascular control (Shi et al., 1993a; Nishiyasu et al., 1999, 2007). Previous experiments reported an increase in stroke volume (SV) and cardiac output (CO) following application of 20–50 mmHg LBPP at rest in supine (Eiken and Bjurstedt, 1987; Shi et al., 1993a) and upright positions (Nishiyasu et al., 1998, 2007) as well as during cycling exercise in upright position (Nishiyasu et al., 1998). This was systematically accompanied by an increase in mean arterial pressure (MAP). Whether such modifications occur during running on a LBPP treadmill remains unknown. Because of their use in clinical settings, it would seem mandatory to understand the cardiovascular implications related to the use of such devices with patients. Furthermore, exercising in a fully upright position (running) compared with exercising while sitting on a saddle (cycling) may have different implications on cardiovascular parameters. A substantial body of literature indeed suggests that—as a results of gravitational forces—cardiovascular parameters such as the volume of blood pooled in the lower extremities, venous return, stroke volume and arterial blood pressure are modified following a transition from sitting to standing (Olufsen et al., 2005).

In order to describe the cardiorespiratory effects of LBPP on running, healthy men took part in two separate protocols that involved standing (EXP-rest) or running (EXP-run) on a treadmill at different levels of LBPP. EXP-rest aimed at investigating the transient responses to LBPP at rest in upright position, while EXP-run allowed us to examine the cardiorespiratory responses at steady-state exercise. We hypothesized that in addition to reducing metabolic demand at a given running speed, LBPP-type bodyweight support may increase CO and/or MAP, thus increasing cardiac work—calculated as the product between the latter parameters—for a given metabolic load.

## Methods

### Participants

Two separate protocols were used in this study. In total, 19 healthy men voluntarily participated to either EXP-rest (*n* = 10; 31 ± 9 y; 179 ± 6 cm; 73 ± 8 kg) or EXP-run (*n* = 9; 27 ± 3 y; 177 ± 6 cm; 68 ± 6 kg). Participants were informed on the experimental procedures and gave written informed consent prior to data collection. None had a history of cardiovascular, respiratory or locomotor disease, were smokers or were taking any medication at the time of experiments. In participants taking part to EXP-run, physical activity readiness was assessed using the French version of the PAR-Q questionnaire (http://www.csep.ca/cmfiles/publications/parq/q-aap.pdf). The experiments were carried out in accordance with the Declaration of Helsinki and approved by the local ethics committee (CERVD; project ID: 2016-00351).

### Experimental protocol

**Figure 1:**
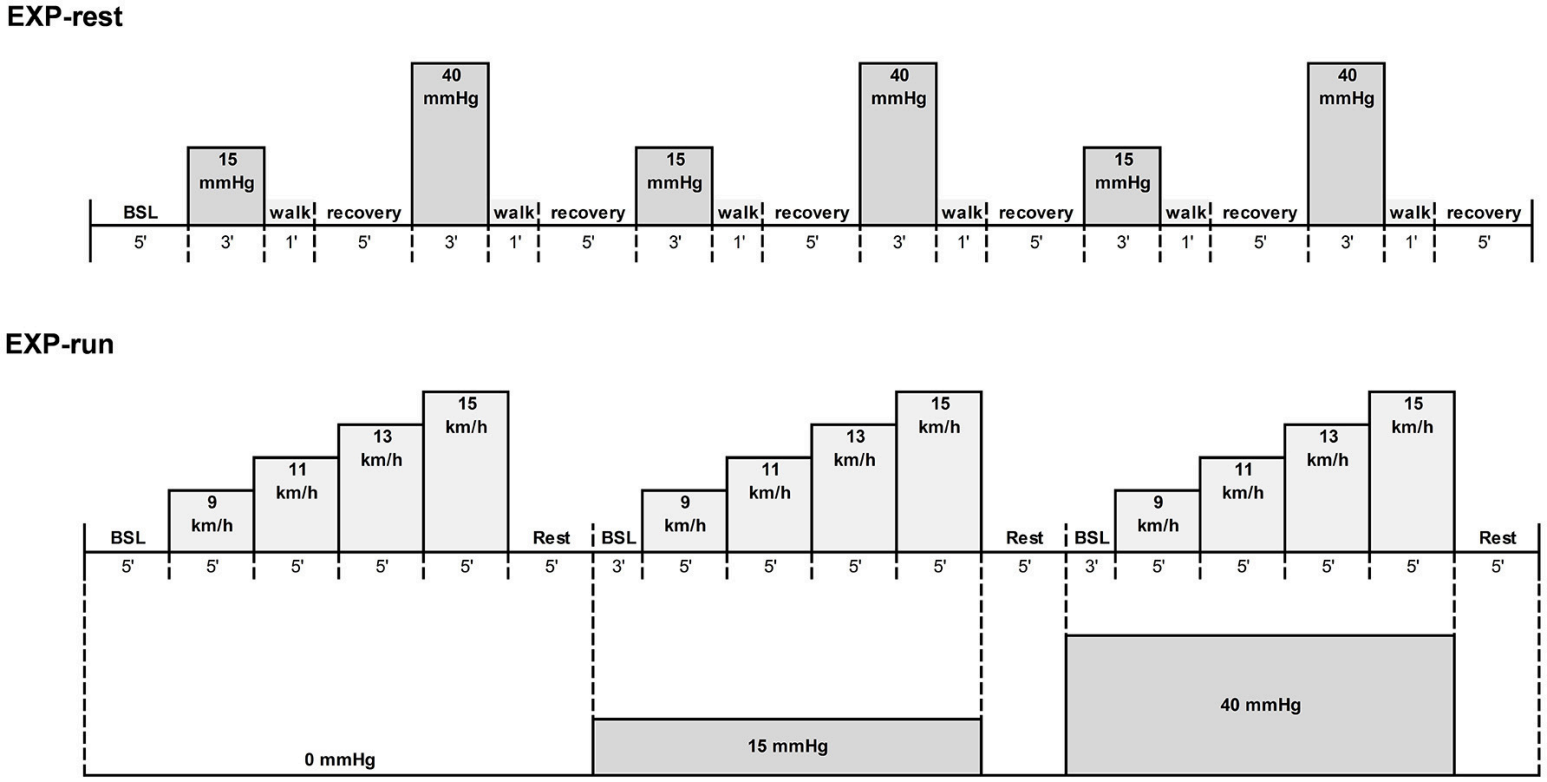
Overview of the experimental protocols. Pressure levels were applied in a randomized order in both experiments. EXP-run: *n* = 9; EXP-rest: *n* = 10.

An overview of the procedure used in both parts (EXP-rest and EXP-run) is presented in Figure 1. In EXP-rest, participants reported to the laboratory on one occasion. After collection of anthropometric measurements (height and weight), participants were sealed into an anti-gravity treadmill (Anti-gravity treadmill Pro-200, Alter-G, Fremont, USA) and the level of bodyweight support was progressively increased to determine the correspondence between bodyweight support (%BW) and LBPP (mmHg) using a calibrated air-pressure sensor (HP206C, Hope Microelectronics, Shenzhen, China) placed inside the flexible chamber. Levels of bodyweight support corresponding to 15 and 40 mmHg were identified for subsequent use during the protocol. Participants were then instrumented on the LBPP-treadmill and asked to stand as still as possible during the entire protocol. After collection of resting values at 0 mmHg LBPP for 5 min, the chamber was inflated at either 15 or 40 mmHg LBPP for 3 min. The pressure was then set back to 0 mmHg and the participant walked at 5 km.h^−1^ for 1 min in order to return to baseline conditions. This sequence was repeated 3 times at 15 mmHg LBPP and 3 times at 40 mmHg LBPP in a randomized balanced order.

In EXP-run, participants reported to the laboratory on two separate occasions interspersed by at least 48 h. During the first visit, after anthropometry and assessment of the correspondence between bodyweight support and pressure, participants were familiarized to the use of the anti-gravity treadmill by running at a self-selected speed at 15 and 40 mmHg LBPP for 10 min each. To estimate participants' ability to complete the experimental protocol, an incremental test to exhaustion at 0 mmHg LBPP was then performed in order to measure maximal aerobic running speed and maximal oxygen consumption with a metabolic cart (Medgraphics CPX, Loma Linda, USA) calibrated with a 3L syringe and gas mixtures of known concentrations of O_2_ and CO_2_ prior to each testing session. After a 5 min warm up at 8 km.h^−1^, running speed was set at 9.7 km.h^−1^ and increased by 0.8 km.h^−1^ every minute until exhaustion was reached (Gojanovic et al., 2012). Peak oxygen consumption (VO_2_peak) was determined as the highest value of VO_2_ reached during the test (30 s average), maximal aerobic velocity was the speed of the last stage completed and maximal heart rate was the highest value recorded during the test (Polar RS400, Polar Electro, Finland). Rate of perceived exertion (RPE) was collected at the end of the test using a 6–20 visual Borg scale.

During the second visit, after instrumentation and warm-up (10 min jogging at 9 km.h^−1^), participants performed three sequences of incremental running exercise on the LBPP-treadmill, each at a different level of LBPP (0, 15, and 40 mmHg) in a balanced randomized order. Each sequence consisted of four consecutive stages of 5 min running at 9, 11, 13, and 15 km.h^−1^ (corresponding to, respectively, 53 ± 5, 64 ± 7, 72 ± 9, and 79 ± 10% peak aerobic running speed at 0 mmHg), interspersed by 5 min of passive recovery and 3 min of quiet standing at the LBPP level of the subsequent sequence.

### Measurements

In EXP-rest, stroke volume (SV), heart rate (HR), cardiac output (CO) mean arterial blood pressure (MAP) and peripheral resistance (PR) were derived with the ModelFlow method using dedicated software (BeatScope Easy, Finapres Medical System, Amsterdam, The Netherlands). Details on the method are presented elsewhere (Wesseling et al., 1993; Imholz et al., 1998).

To estimate baroreflex sensitivity (BRS), even though the sequence method that consists in regressing three or more successive increasing/decreasing RR-intervals (RRI) on the corresponding increasing/decreasing values of systolic blood pressure (SBP) is considered as the reference (La Rovere et al., 2008), it may be problematic when only a few sequences (or even none) are present in the recording. We therefore used the ratio between the standard deviations RRI/SBP, a method shown to be robust and consistent (Bernardi et al., 2010). Prior to computation of this ratio before (−2.5 s to LBPP) and after (LBPP to +2.5 s) application of LBPP, RRI, and SBP data were resampled at 4 Hz and respiratory sinus arrhythmia was removed from the resampled RRI and SBP signals using adaptive interference cancelation, as suggested by Tiinanen et al. (2008).

In order to apprehend blood shifts from the lower body, tissue oxygenation of the left thigh and calf was monitored in 7 participants by using near-infrared spectroscopy (NIRS) (Oxymon Mk III, Artinis, The Netherlands). This allowed the measurement of changes in blood concentration in oxygenated and deoxygenated hemoglobin ([O_2_Hb] and [HHb], respectively), as well as total hemoglobin (Hbt). Two NIRS probes were fixed to the skin of the left vastus lateralis (15 cm above the upper edge of the patella) and left gastrocnemius (5 cm above the middle of the musculotendinous junction with the Achilles tendon, and 5 cm lateral from this point), with the light emitters and transmitters aligned with the vertical axis of the leg. The three transmitting diodes operated at wavelengths of 760 and 850 nm respectively. The distance between the light emitters and transmitters was set at 4.5 cm.

In EXP-run, SV, HR, and CO were continuously and noninvasively monitored using bioimpedance cardiography (Physioflow Enduro, Manatec Biomedical, Paris, France). This method has been described in detail elsewhere (Charloux et al., 2000). Briefly, it couples an estimation of SV from variations of transthoracic impedance during systole with a measurement of HR from ECG to compute beat-to-beat CO. Oxygen uptake was measured breath-by-breath using a respiratory gas analyser (Medgraphics CPX, Loma Linda, USA). Beat-to-beat systolic (SBP) and diastolic (DBP) blood pressure were continuously and noninvasively measured using a digital sphygmomanometer (CNAP monitor 500HD, CNSystems Medizintechnik AG, Graz, Austria) on the middle finger of the left hand in 6 subjects. MAP was calculated by mathematical integration of the continuous BP trace over the last minute of each stage. PR was calculated by dividing MAP by CO. To minimize signal noise due to vibrations or movements, participants positioned their left arm in a custom-built arm rest mold attached to the treadmill and relaxed their fingers as much as possible during the protocol.

Data were continuously acquired at a frequency of 200 Hz using an analog-to-digital converter (ML880 PowerLab 16/30, ADInstruments, Bella Vista, Australia) coupled with commercially available software (LabChart version 7.2, ADInstruments, Bella Vista, Australia), and stored in a computer for later analysis.

### Data analysis

In EXP-rest, haemodynamic data, breath-by-breath gas exchange data and NIRS data were resampled at 200 Hz and aligned prior to analysis with MATLAB (v15.0, The Mathworks, Natick, MA, USA). Baseline and steady-state values were then calculated by averaging the data collected during the last 30 s preceding the application, respectively the release of LBPP. The transient response was assessed as the maximal value observed within the first minute of LBPP application for CO, SV, and VO_2_ while the minimal value was selected for end-tidal oxygen tension (P_ET_O_2_) and HR. Since both PR and MAP signals showed a biphasic shape following application of LBPP, both the maximal and the minimal values occurring within the first minute were considered in the analysis. Transient Hbt (calf and quadriceps) was assessed by averaging 30 samples (0.15 s) around the first minimal value following LBPP. Hbt data are expressed as the difference from baseline value.

In EXP-run, data collected during the last minute of each stage were averaged to obtain steady-state measurements of HR, SV, CO, MAP, and PR. Baseline data at each level of pressure were obtained by averaging the last minute of the corresponding resting period.

### Statistics

In order to detect any significant effect of pressure and speed on the hemodynamic and metabolic responses to submaximal exercise, a two-way repeated measures ANOVA was performed in EXP-run using Bonferroni correction, while a one-way repeated measures ANOVA was used in EXP-rest to assess the effect of pressure on hemodynamic parameters. Data were analyzed using GraphPad Prism, version 6.01 (GraphPad Software Inc., La Jolla, CA, USA). Significance was set at *P* < 0.05. Normality of data distributions was assessed using the Kolmogorov-Smirnov test. Only the MAP response dataset in EXP-rest was found not normally distributed (*P* < 0.05). In this case, a Friedman test was used to detect any significant effect of pressure.

## Results

### Transient response at rest

**Figure 2:**
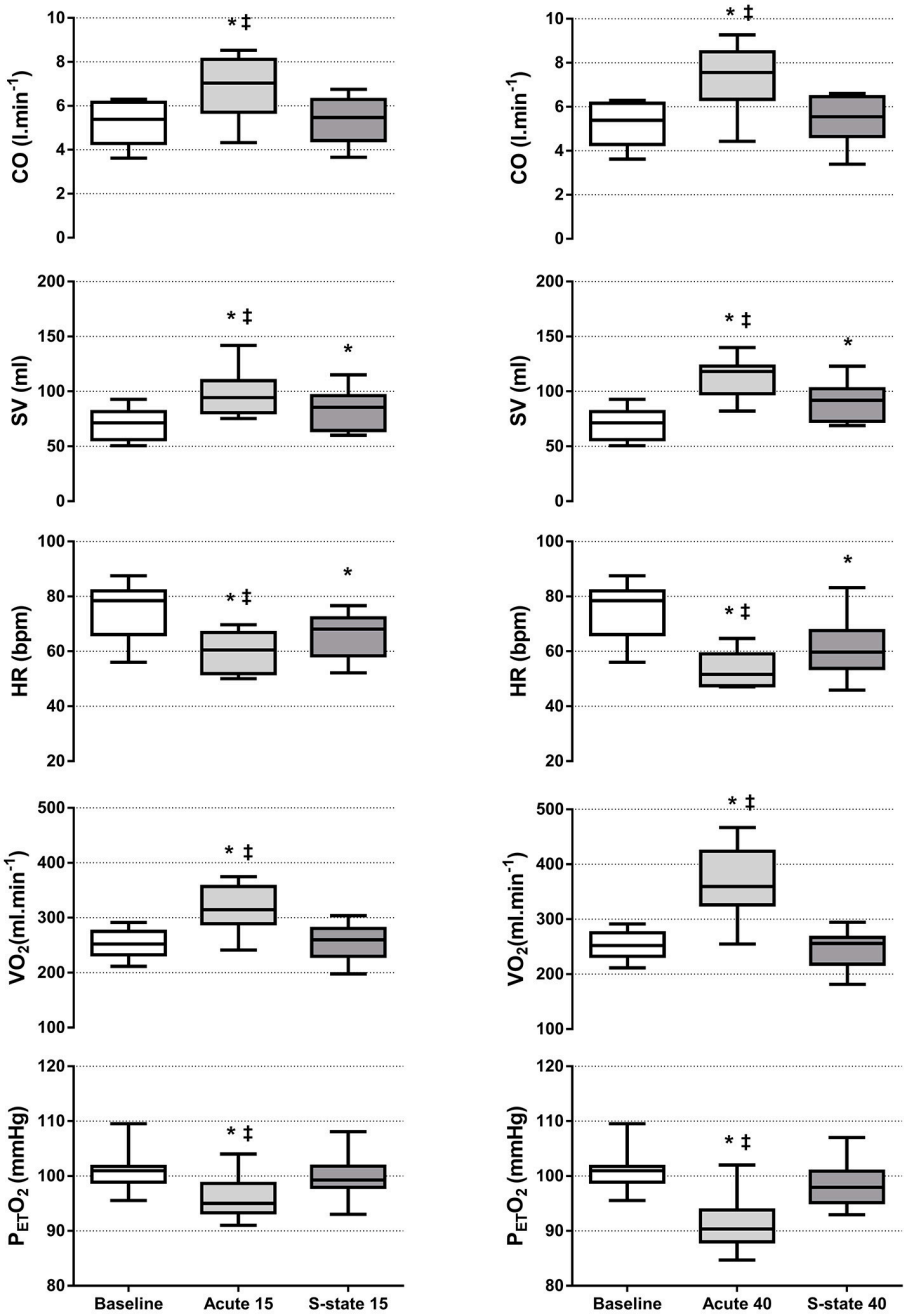
Changes in cardiorespiratory parameters over the LBPP periods during EXP-rest. Boxes extend from the 25th to 75th percentiles, upper and lower whiskers represent maximum and minimum values, respectively. *N* = 10; ^*^different from baseline and steady-state (*P* < 0.05); ‡different from steady-state (*P* < 0.05). **(Left)** LBPP 15 mmHg. **(Right)** LBPP 40 mmHg. Acute, within first minute of LBPP; S-state, steady-state after three minutes of LBPP.

**Figure 3:**
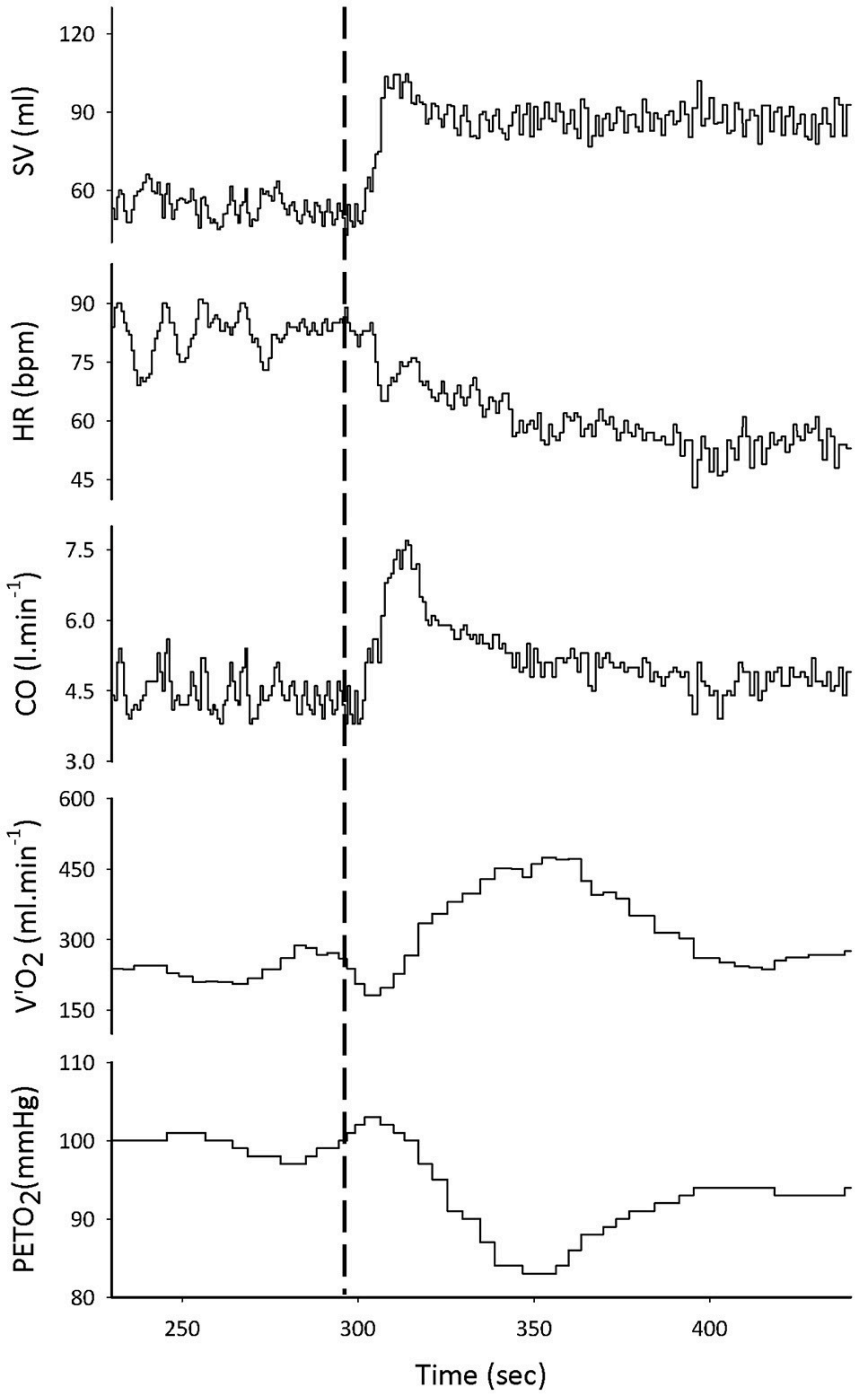
Representative example of tracings taken over the EXP-rest protocol. The dashed line marks the application of LBPP. From top to bottom: stroke volume (SV), heart rate (HR) and cardiac output (CO), oxygen consumption (VO_2_), and end-tidal PO_2_ (P_ET_O_2_).

Cardiorespiratory responses to transient LBPP at rest are shown on Figure 2. Within the first seconds of LBPP, SV increased (by +29 ± 13 and +42 ± 15 ml, corresponding to a +41 and +60% change from baseline, at 15 and 40 mmHg, respectively (*p* < 0.05)) while HR decreased [by −15 ± 6 (−20%) and −22 ± 9 (−29%) bpm (*p* < 0.05)], resulting in a transitory increase in CO [by +1.6 ± 1.0 (+32%) and +2.0 ± 1.0 (+39%) l.min^−1^ (*p* < 0.05)]. This was accompanied by a transitory decrease in P_ET_O_2_ [by −5 ± 3 (−5%) and −10 ± 4 (−10%) mmHg (*p* < 0.05)] and a transitory increase in VO_2_ [by +66 ± 53 (+26%) and +116 ± 64 (+46%) ml.min^−1^ (*p* < 0.05)]. Those effects were significantly greater at 40 mmHg compared to 15 mmHg, except for CO. Dynamic changes in these parameters are illustrated in Figure 3.

After 3 min of LBPP, HR [75 ± 10 vs. 59 ± 8 vs. 52 ± 11 bpm (*P* < 0.05)] and SV remained altered [70 ± 14 vs. 83 ± 18 vs. 90 ± 18 ml (*P* < 0.05)], while their product resulted in a return of CO toward baseline values [5.2 ± 1.0 vs. 5.4 ± 1.0 vs. 5.4 ± 1.1 l.min^−1^ at 0, 15, 40 mmHg LBPP, respectively (*P* > 0.05)]. The initial changes observed in VO_2_ were also progressively normalized at both levels of pressure [254 ± 25 vs. 256 ± 34 vs. 245 ± 34 ml.min^−1^ at 0, 15, 40 mmHg LBPP, respectively (*P* > 0.05)] and in P_ET_O_2_ at 15 mmHg [101 ± 4 vs. 96 ± 4 mmHg at 0 and 15 mmHg, respectively (*P* > 0.05)] but not at 40 mmHg where it remained altered compared to baseline [101 ± 4 vs. 91 ± 5 mmHg at 0 and 40 mmHg, respectively (*P* < 0.05)].

**Figure 4:**
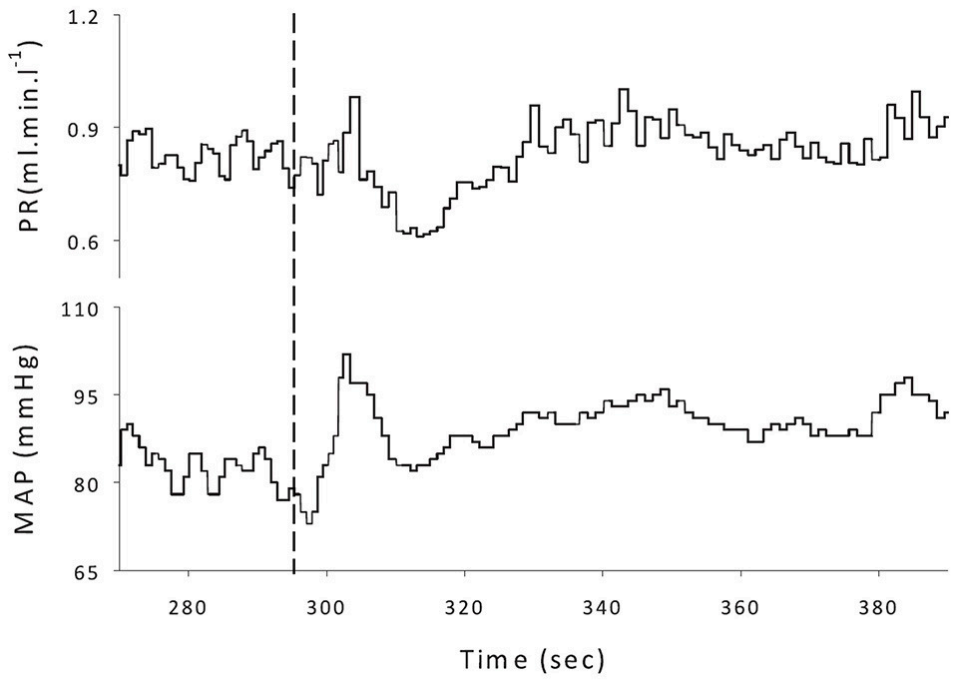
Representative example of tracings taken over the EXP-rest protocol. The dashed line marks the application of LBPP. **(Top)** Peripheral resistance (PR); **(Bottom)** mean arterial pressure (MAP).

Upon the rise in LBPP, MAP showed an immediate increase that reached significance at 40 mmHg [+16 ± 5 (+18%) mmHg, (*P* < 0.05)] but not at 15 mmHg [+15 ± 7 (+16%) mmHg, (*P* = 0.056)]. This was followed by a rapid drop restoring baseline values at the end of the LBPP periods [92 ± 13 vs. 95 ± 11 vs. 97 ± 16 mmHg at 0, 15, and 40 mmHg, respectively (*P* > 0.05)]. PR also showed an immediate increase [by +0.26 ± 0.17 (+22%) and +0.26 ± 0.16 (+22%) mmHg.min.l^−1^ at 15 and 40 mmHg LBPP, respectively (*P* < 0.05)]. This was followed by a rapid drop reaching significantly lower values compared to baseline [by −0.29 ± 0.23 (−25%) and −0.36 ± 0.23 (−30%) mmHg.min.l^−1^ at 15 and 40 mmHg LBPP, respectively (*P* < 0.05)] before a second, slower rise restored baseline values at the end of the LBPP periods [1.18 ± 0.37 vs. 1.17 ± 0.35 vs. 1.23 ± 0.44 mmHg.min.l^−1^ at 0, 15, and 40 mmHg, respectively (*P* > 0.05)]. Figure 4 illustrates the typical pattern of response to LBPP in MAP and PR.

**Figure 5:**
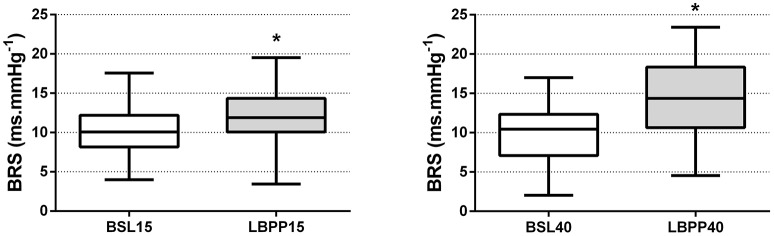
Baroreflex sensitivity (BRS) at the onset of LBPP. Baroreflex sensitivity after application of LBPP at 15 (LBPP15) and 40 (LBPP40) mmHg compared to the corresponding baselines at 0 mmHg (BSL15 and BSL40, respectively) (*n* = 10). BRS is expressed as the ratio between standard deviations RRI/SBP. Boxes extend from the 25th to 75th percentiles, upper and lower whiskers represent maximum and minimum values, respectively. ^*^Different from baseline (*P* < 0.05).

As shown in Figure 5, after application of 15 and 40 mmHg LBPP, BRS increased significantly [by +1.8 ± 1.6 (+18%) and +4.6 ± 3.7 (+47%) ms.mmHg^−1^ at 15 and 40 mmHg LBPP, respectively, *P* < 0.05] compared to the corresponding baseline, with a greater effect size at 40 mmHg compared to 15 mmHg (0.42 vs. 0.91, respectively).

During the transient phase following the application of LBPP, NIRS measurements showed a significant decrease in Hbt at 40 mmHg LBPP compared to baseline, both on the calf [0.26 ± 0.14 vs. 0.13 ± 0.11 μmol at 0 and 40 mmHg, respectively (*P* < 0.05)] and on the quadriceps [0.14 ± 0.15 vs. −0.01 ± 0.16 μmol (*P* < 0.05)].

### Exercise steady state

**Figure 6:**
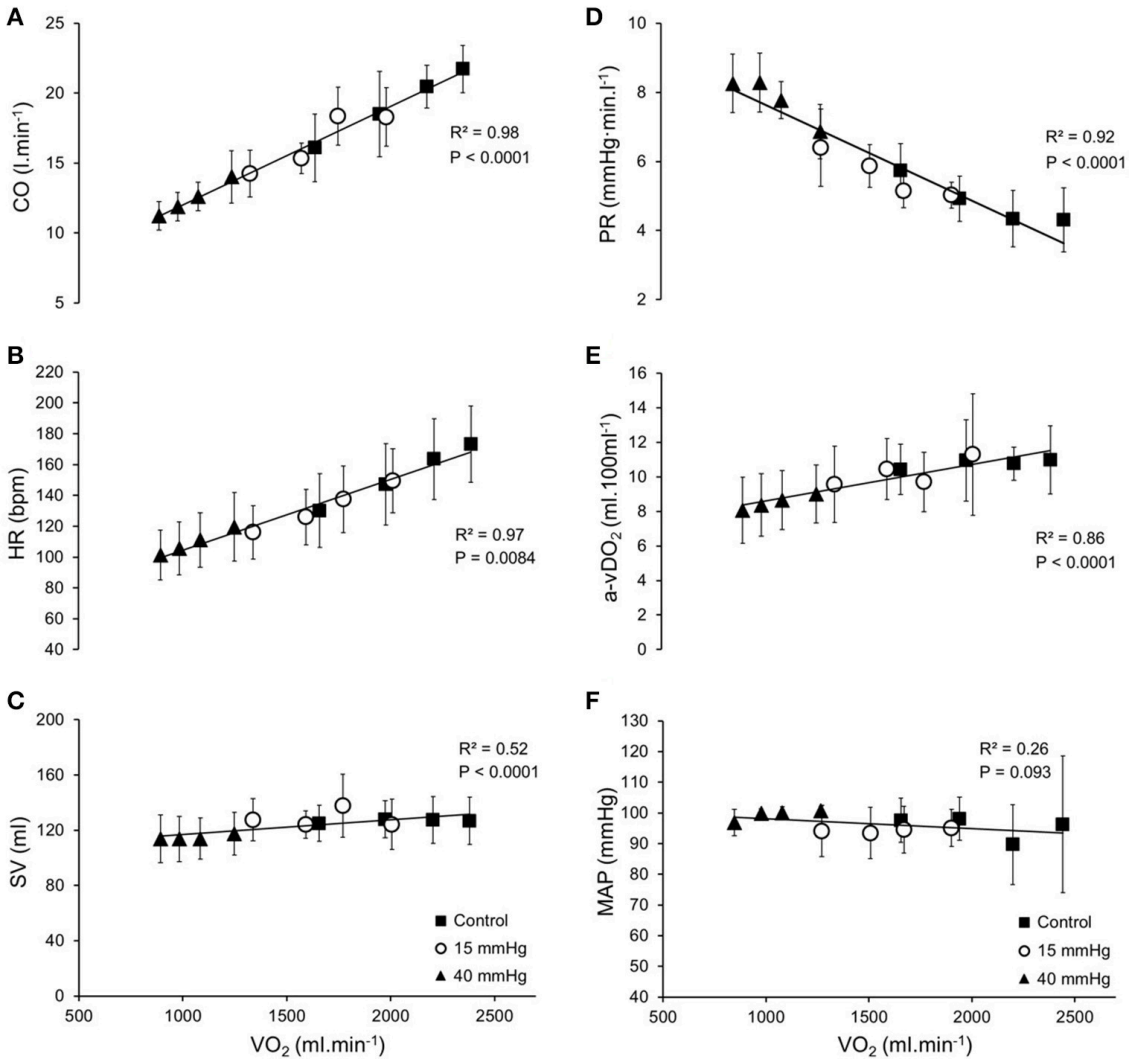
Cardiorespiratory parameters at exercise steady-state. Average steady-state values (±SD) at each pressure level (▲: 40 mmHg; ○: 15 mmHg; ■: control) as a function of oxygen uptake (VO_2_) (*n* = 9); **(A)** Cardiac output, CO; **(B)** Heart rate, HR; **(C)** Stroke volume, SV; **(D)** Peripheral resistance, PR; **(E)** Systemic arteriovenous oxygen difference, a-vDO_2_; **(F)** Mean arterial pressure, MAP. Systemic arteriovenous oxygen difference (a-vDO_2_) was calculated as oxygen uptake (VO_2_) divided by cardiac output (CO) for each level of lower body positive pressure and speed.

Cardiorespiratory parameters at exercise steady-state obtained at all LBPP levels and running speeds are shown in Figure 6. At the steady-state of each running speed, when compared to the control condition (i.e., 0 mmHg), the application of 15 or 40 mmHg LBPP led to a significant decrease in CO [by an average of −2.6 ± 0.8 l.min^−1^ (−14%) and −6.8±1.4 l.min^−1^ (−35%), respectively (*P* > 0.05)], HR [by −21 ± 5 bpm (−14%) and −44 ± 12 bpm (−29%) (*P* > 0.05)] and VO_2_ [by −378 ± 48 ml.min^−1^ (−18%) and −1,002 ± 172 ml.min^−1^ (−48%) (*P* > 0.05)]. Similarly, a decrease in SV was observed at 40 mmHg LBPP compared to 0 and 15 mmHg LBPP, [by −13 ± 2 ml and −16 ± 7 ml, respectively (*P* < 0.05)] except for the last stage of exercise (15 km.h^−1^) were the decrease was found not significant. MAP remained unchanged at all speeds in all conditions, while PR increased at all speeds at 40 mmHg LBPP when compared to 0 and 15 mmHg LBPP [by an average of +3.0 ± 0.5 mmHg.min.l^−1^ (+16%) and +2.2 ± 0.4 mmHg.min.l^−1^ (+61%), respectively (*P* < 0.05)].

When CO was expressed as a function of VO_2_ (representing metabolic load), linear relationships were found in all conditions, with similar slopes [6.6 ± 3.6 vs. 7.4 ± 3.9 vs. 7.3 ± 2.2 ml.min^−1^/ml.min^−1^ at 0, 15, and 40 mmHg LBPP, respectively (*P* > 0.05)]. When data points were plotted together regardless LBPP level, relationships were linear, as shown in Figure 6A [mean *R*^2^ = 0.84 ± 0.13 for each participant taken individually and *R*^2^ = 0.98 when plotting average data points at the steady-state of each stage, for all LBPP levels (*P* < 0.05)].

Similarly, PR vs. VO_2_ relationships in the three conditions were linear with slopes that were not different [1.9 ± 1.0 vs. 2.2 ± 1.1 vs. 3.1 ± 2.2 mmHg.min.l^−1^/ml.min^−1^ at 0, 15, and 40 mmHg LBPP, respectively (*P* > 0.05), mean *R*^2^ = 0.84 ± 0.09; *R*^2^ = 0.92], as shown on Figure 6D.

## Discussion

The main findings of this study were that (1) LBPP in standing position transiently affects cardiorespiratory integration at the onset of pressure application; (2) at a given LBPP, once reaching steady-state exercise, the cardiorespiratory load is reduced proportionally to the lower metabolic demand resulting from the body weight support; (3) the balance between cardiovascular response, oxygen delivery to the exercising muscles and blood pressure regulation is maintained at exercise steady-state; and (4) changes in baroreflex sensitivity may be involved in the regulation of cardiovascular parameters during LBPP.

### Cardiorespiratory responses to LBPP at rest

In order to investigate the effects of LBPP-treadmills on venous return and cardiorespiratory regulation without confounding from exercise-induced activation of the muscle pump and the reduction in metabolic demand resulting from bodyweight support during locomotion, we asked the participants to stand still on the LBPP-treadmill, while various levels of LBPP were applied. Within the first seconds following the application of LBPP, we observed a significant increase in SV at both 15 and 40 mmHg LBPP (Figure 2). These changes remained significant throughout the LBPP period, although a gradual decline was observed with time. According to previous research, this reflects increased cardiac filling secondary to the LBPP induced displacement of blood from the lower limbs toward the central circulation (Jennings et al., 1986a; Eiken and Bjurstedt, 1987; Ng et al., 1987; Shi et al., 1993a,b; Nishiyasu et al., 1998, 2007). The immediate decreases in Hbt that we observed with the calf and thigh NIRS probes at 40 mmHg corroborate this contention. The initial increase in SV resulted in an immediate increase in CO that remained significant only transitorily, before returning to baseline values (Figure 2). This restoration of CO was the consequence of a decrease in HR, presumably caused by an activation of the baroreflex upon stimulation of arterial and cardiopulmonary baroreceptors resulting from the increased venous return (Ng et al., 1987; Nishiyasu et al., 1998, 2007). In addition to its effect on HR, activation of the baroreflex may have induced peripheral vasodilation, explaining the restoration of PR after an initial increase following the inflation of the chamber (Table 1).

**Table 1:** Transient and steady-state MAP and PR responses.

**Parameters**	**Baseline**	**15 mmhg**			**40 mmHg**		
		**Max**	**Min**	**Steady-state**	**Max**	**Min**	**Steady-state**
MAP [mmHg]	92.41 ± 12.53	106.60 ± 15.00	81.70 ± 7.22	95.23 ± 10.82	108.93 ± 14.19^*^^†^	81.77 ± 11.01	97.37 ± 15.49
PR [mmHg.min.l^−1^]	1.18 ± 0.37	1.43 ± 0.47^*^	0.88 ± 0.27^*^	1.17 ± 0.35	1.45 ± 0.42^*^	0.82 ± 0.28^*^	1.23 ± 0.44

*Transient response correspond to the maximal and minimal values within the first minute of the LBPP period, while steady-state responses was calculated as the average of the last minute (n = 10)*.

* Different from baseline and steady-state (P < 0.05);

† *different from 15 mmHg (P < 0.05)*.

Interestingly, analysis of BRS indicated a significant increase both at 15 and 40 mmHg LBPP (Figure 5), with a greater effect at 40 mmHg. Previous studies that investigated the influence of LBPP on baroreflex activity showed a resetting of its cardiac component with (Eiken et al., 1992) or without (Gallagher et al., 2001b) changes in sensitivity. These changes were attributed to the activation of both the mechanical and the metabolic component of the exercise pressor reflex (EPR) (Fadel and Raven, 2012). Although our results suggest that LBPP modified baroreflex sensitivity, it should be noted that the method used in our study does not allow the determination of the full stimulus-response relationship. However, the sequence method—an equivalent of the technique used in this study (Bernardi et al., 2010)—has been shown to report changes in operating point gain of the carotid HR-baroreflex curve (Ogoh et al., 2005). Alterations in BRS observed in our study following LBPP may therefore explain the restoration of CO, MAP and PR with time despite LBPP being maintained at the same level, reflecting the achievement of a new equilibrium reached through baroreflex resetting. In addition, although HR remained significantly altered until deflation of the chamber, it is interesting to note that a gradual restoration toward baseline values was observed with time (Figure 2). In support, Yanagida et al. (2014) showed that mild hypergravity reduces spontaneous baroreflex sensitivity. They posited that one of the possible mechanisms for the reduction in cardiac baroreflex sensitivity was that cardiopulmonary receptor deactivation by central hypovolemia interacts with cardiac baroreflex center. It appears likely that in our experimental set-up the opposite occurred.

Our results showing cardiovascular alterations following LBPP are in contradiction with earlier studies that reported no significant effects of LBPP on SV or HR (Jennings et al., 1986b; Williamson et al., 1994a,b; Nishiyasu et al., 1998, 2007; Fu et al., 2000; Gallagher et al., 2001a). However, it should be noted that these experiments were conducted in supine participants, which may explain the discrepancies with our results. Notably, in their studies comparing the cardiovascular responses to LBPP in supine vs. upright participants, Nishiyasu et al. (1998, 2007) suggested that the greater pooling of blood in the lower body caused by gravitation in the upright posture (≈600 ml) explained the more pronounced effect of LBPP on blood transfer toward the right heart while upright, which would explain that SV increased significantly in this position, but remained unchanged in the supine position. The unchanged HR seen in supine participants—in contrast with the decrease observed in upright participants—was attributed to a lower activation of the baroreceptors secondary to the lower impact of LBPP on blood displacement.

The transitory increase in CO observed in our study upon application of LBPP was accompanied by a transitory rise in VO_2_ measured at the mouth that was restored shortly after (Figure 2). Because the participants were in resting conditions and were required to stand still during the protocol, this increase in VO_2_ could not be the result of an increase in metabolic demand. Rather, this phenomenon reflects a transient translocation of blood from the lower limbs to the right heart caused by LBPP, increasing in turn pulmonary blood flow and lung capillary recruitment. The consequent increase in deoxygenated hemoglobin availability at the alveolar-capillary interface likely increased oxygen transfer into the pulmonary blood, thus decreasing the partial pressure of oxygen at the alveolar level, which was interpreted as an increase in VO_2_ uptake by the gas analyser. The simultaneous transient decrease in P_ET_O_2_ observed with the increase in VO_2_ would support this interpretation. This observation confirms the influence of changes in venous return on the measurement of VO_2_ (Barstow, 1994). Notably, the immediate rapid increase in VO_2_ observed at the onset of exercise is thought to be primarily due to an increase in cardiac output in response to exercise, rather than reflecting changes in metabolic state in the exercising muscles (Wasserman et al., 1974). In our study, the increased venous return due to LBPP might have had—to some extent—a similar effect.

Assuming that the increase in VO_2_ measured at the mouth was due to a surge of blood coming from the lower body, the volume of additional blood flowing through the pulmonary circulation was estimated as the mathematical integration of the VO_2_ signal from the application of LBPP to the point where VO_2_ returned to baseline values—which allowed to estimate the total excess VO_2_ in milliliters over time—divided by 40 ml of oxygen transferred through the alveolar capillary barrier per liter of blood (Guyton and Hall, 2006). This calculation yielded an estimate of ~ 2L of additional blood flowing through the pulmonary circulation over 1 min.

### Cardiorespiratory responses to LBPP during exercise

Cardiorespiratory parameters were assessed at the steady-state of running exercise performed at 0, 15, and 40 mmHg. On average, the highest workload achieved during the experimental protocol (i.e., 15 km.h^−1^ at 0 mmHg LBPP) corresponded to 79% of the VO_2_max obtained during the incremental test to exhaustion. Because this lies close to the lower boundary of the severe exercise intensity domain (Poole and Jones, 2012) and therefore to ensure that a steady state was reached at this intensity, the stability of the VO_2_ and CO responses was assessed during the last minute of the stage by comparing the average value during the first and the last 10 s of the last minute of the stage. Percent changes for VO_2_ (4%) and CO (8%) between those values confirmed that these responses were reasonably stabilized at the end of the stage.

During exercise, we found that LBPP significantly decreased HR and VO_2_ at steady-state at all tested running speeds and that this effect was more pronounced at 40 mmHg LBPP (Figure 6B). These results are in accordance with previous studies using LBPP-type anti-gravity treadmills at various levels of bodyweight support (i.e., various levels of LBPP) (Danielsson and Sunnerhagen, 2000; Cutuk et al., 2006; Ruckstuhl et al., 2010; Hoffman and Donaghe, 2011; Sota et al., 2013). Nishiyasu et al. (1998) observation of unchanged VO_2_ following application of LBPP during cycling exercise, supports the idea that the reduced VO_2_ found in our study was due to the weight unloading effect of LBPP rather than some effect on venous return through vessel compression. In addition, we demonstrated for the first time that CO and SV were also significantly reduced by LBPP at a given running velocity (Figures 6A,C, respectively). These results suggest that the LBPP-induced bodyweight support proportionally adapts the cardiovascular response to the reduced metabolic load of exercise at a given speed. Whether LBPP *per se* would affect venous return and therefore modify cardiorespiratory responses to running exercise otherwise than through weight unloading remained to be determined, which is what we attempted. Since the diminished metabolic demand due to the unloading made analysis of CO as a function of running speed impossible, CO was expressed as a function of VO_2_. Results show that this relationship remained linear, with similar slopes regardless the level of pressure (Figure 6A). In accordance with our results, several authors previously observed that CO remained unchanged during exercise with LBPP at similar metabolic loads (Williamson et al., 1994a; Gallagher et al., 2001a; Sperlich et al., 2011). Hoffman and Donaghe (2011) reported invariant HR and VO_2_ relationships in healthy participants running on a LBPP-treadmill with 25 and 50% of bodyweight support. They explained their results by assuming that the action of the muscle pump, along with other exercise-induced cardiovascular responses might have overridden the effect of LBPP on venous return. Since the muscle pump produces a driving force of 90 mmHg (Gallagher et al., 2001a) and that we used LBPP levels well below this value, it is likely that any potential effect on venous return produced by LBPP below 90 mmHg would be overridden by the effect of muscle contractions during exercise. The fact that the CO vs. VO_2_ relationship observed in our study remained linear regardless the level of LBPP might therefore be explained in part by this contention.

However, that LBPP failed to produce a further translocation of blood between the lower body and the central circulation, once steady exercise was reached, is in contradiction with other studies that reported increased CO during exercise performed at similar levels of LBPP, and that attributed this observation precisely to an elevated venous return under the action of LBPP (Ng et al., 1987; Nishiyasu et al., 1998). These discrepancies may be explained by the differences in exercise modalities between studies, in which the muscle pump of the lower limbs may have been either inoperative (arm cranking) or minimized (cycling at low intensity) compared to running. Moreover, Eiken and Bjurstedt (1987) attributed the rise in CO observed in their study to a feedforward mechanism signaled by muscle metaboreceptors aiming to restore arterial blood flow restriction caused by LBPP. Given the absence of a concomitant increase in HR in our study, it seems unlikely that this phenomenon did occur.

In addition to these results, we observed that MAP and PR remained similar at all speeds and were unaffected by LBPP. Although several studies found an increase in MAP during exercise performed with LBPP, most attributed this rise to either a concomitant increase in CO (Eiken and Bjurstedt, 1987; Ng et al., 1987) or an activation of muscle mechanoreceptors (*mechanoreflex*) following LBPP (Williamson et al., 1994a; Nishiyasu et al., 1998; Gallagher et al., 2001a,b). As mentioned above, we did not observe such an increase in CO, which would therefore explain the unchanged MAP. As for the mechanoreflex, the absence of a concomitant increase in HR suggests that the methodology used in our study prevented such mechanism to occur. It should be noted that none of the studies mentioned above were conducted in upright participants, which might also have had an impact on the MAP response (Nishiyasu et al., 1998). In line with this, Cutuk et al. (2006) found no significant differences in MAP when LBPP was applied in participants walking on an anti-gravity treadmill, a procedure quite similar to the methodology used in our study. When PR was expressed as a function of VO_2_, the relationship remained linear regardless the level of LBPP (Figure 6D). This would suggest that cardiovascular afterload was not affected by LBPP and that the decrease in PR observed with the increase in metabolic load was due solely to the effect of speed and bodyweight support. Given that the cardiovascular parameters were unchanged at exercise steady-state, it seems that the increase in baroreflex sensitivity observed at rest at the onset of LBPP did not occur in these conditions. This may be explained by a resetting of the baroreflex described during physical exercise (Fadel and Raven, 2012) that may have overridden the LBPP-induced increase in BRS.

### Limitations

Some limitations to our study design should be acknowledged. Firstly, we used the Modelflow® method from fingertip pulse pressure measurement to estimate CO during EXP-rest. Although it has been reported that this technique allows satisfactory estimates of CO in resting conditions (Jansen et al., 1990; Antonutto et al., 1994), some authors have questioned its accuracy based on the fact that the model from which CO is computed uses standard values of aortic properties which differ from those of finger arteries to reconstruct aortic blood flow (Azabji Kenfack et al., 2004). Since accuracy of the measurement during exercise has been questioned by several authors (Houtman et al., 1999; Azabji Kenfack et al., 2004), we restricted the use of this technique to the assessment of CO in resting conditions (i.e., during EXP-rest).

Secondly, during EXP-run, CO was assessed by impedance cardiography using Physioflow®. The accuracy and reliability of impedance cardiography for the measurement of CO has been debated, especially with regard to absolute values (Boer et al., 1979). Because of the potential errors introduced by several factors including electrode placement, perspiration and movements that would impair the measurement of basal thoracic impedance (Z_0_) needed for the calculation of SV. To deal with this issue, technical developments have been made to avoid the measurement of Z_0_ and to use the variations of impedance signal waveform to estimate SV. This technique has been shown to provide a reasonable evaluation of cardiac output at rest as well as during exercise when compared to the “direct” Fick method (Charloux et al., 2000). To increase signal stability we designed the experiment to be completed during a single visit, with repeated measures, to limit intra- and inter-individual errors due to electrode placement and anthropometric characteristics, respectively. Also, we did not base our analysis on absolute CO values, which have been the object of most of the criticisms.

Thirdly, during EXP-rest, we used changes in Hbt measured by NIRS to appraise blood displacement from the lower limb, in 7 subjects. Although Hbt and blood volume have been shown to correlate well (De Blasi et al., 1994), this parameter does not present a direct measurement of venous return.

Fourthly, it should be acknowledged that our analysis did not take into account local mechanisms regulating vasodilation at the muscle level, which are known to influence CO regulation during exercise (González-Alonso et al., 2008). Whether the levels of LBPP used in our study influenced these local mechanisms remains unknown.

Finally, it should be acknowledged that given the small sample sizes used in both EXP-rest (*n* = 10) and EXP-run (*n* = 9), caution must be taken when interpreting the results obtained.

## Conclusion

Our results suggest that LBPP applied on anti-gravity treadmills reduces the cardiorespiratory load proportionally to the reduced metabolic demand resulting from the body weight support during exercise. Initial cardiovascular changes observed at rest indicate that LBPP increases venous return, in turn increasing baroreflex sensitivity but that these effects are abolished during steady-state exercise, presumably due to the action of the muscle pump overriding the effect of LBPP on venous return and to the associated exercise-induced baroreflex resetting. Further investigations are required to confirm these results in other populations including patients enrolled in rehabilitation programmes.

## Author contributions

BU, FS, and BK conceived and designed the research; BU and FS performed the experiments; BU, FS, and J-MV analyzed the data; BU, FS, BK, and J-MV interpreted the results; BU and FS drafted the manuscript and prepared figures; BU, FS, BK, and J-MV edited and revised the manuscript and approved the final version.

### Conflict of interest statement

The authors declare that the research was conducted in the absence of any commercial or financial relationships that could be construed as a potential conflict of interest.
